# Distribution of lineages and type II toxin-antitoxin systems among rifampin-resistant *Mycobacterium Tuberculosis Isolates*

**DOI:** 10.1371/journal.pone.0309292

**Published:** 2024-10-24

**Authors:** Maryam Shafipour, Abdolmajid Mohammadzadeh, Pezhman Mahmoodi, Mahdi Dehghanpour, Ezzat Allah Ghaemi

**Affiliations:** 1 Department of Pathobiology, Faculty of Veterinary Medicine, Bu-Ali Sina University, Hamedan, Iran; 2 Razi Vaccine and Serum Research Institute, Agricultural Research, Education and Extension Organization (AREEO), Karaj, Iran; 3 Infectious Diseases Research Center, Golestan University of Medical Sciences, Gorgan, Iran; University of Padova, ITALY

## Abstract

Type II toxin-antitoxin systems such as *mazEF3*, *vapBC3*, and *relJK* play a role in antibiotic resistance and tolerance. Among the different known TA systems, *mazEF3*, *vapBC3*, and *relJK*, which are type II systems, have specific roles in drug resistance. Therefore, the aim of this study was to investigate the mutations in these genes in sensitive and resistant isolates of *Mycobacterium tuberculosis*. Thirty-two rifampin-resistant and 121 rifampin-sensitive *M*. *tuberculosis* isolates were collected from various regions of Iran. Lineage typing was performed using the ASO-PCR method. Mutations in the *rpoB* gene were analyzed in all isolates by MAS-PCR. Furthermore, mutations in the *mazEF3*, *relJK*, and *vapBC3* genes of the type II toxin system were assessed through PCR sequencing. These sequences were analyzed using COBALT and SnapGene 2017, and submitted to the GenBank database. Among the 153 *M*. *tuberculosis samples*, lineages 4, 3 and 2 were the most common. Lineage 2 had the highest rate of rifampin resistance. Mutations in *rpoB531* were the most frequent in resistant isolates. Examination of the toxin-antitoxin system showed that rifampin-resistant isolates belonging to lineage 3 had mutations in either the toxin or antitoxin parts of all three TA systems. A mutation in nucleotide 195 (codon 65) of *mazF3* leading to an amino acid change from threonine to isoleucine was detected in all rifampin-resistant isolates. *M*. *tuberculosis* isolates belonging to lineage 2 exhibited the highest rifampin resistance in our study. Identifying the mutation in *mazF3* in all rifampin-resistant isolates can highlight the significance of this mutation in the development of drug resistance in *M*. *tuberculosis*. Expanding the sample size in future studies can help develop a new method for identifying resistant isolates.

## Introduction

Several genetic modules are involved in antibiotic resistance. Transcriptomic analysis of antibiotic-resistant *Mycobacterium tuberculosis* (*M*. *tuberculosis*) isolates revealed that in pathogenic *Mycobacteria*, Toxin-Antitoxin (TA) systems are significantly expressed [1, 2]. To date, approximately 90 putative TA systems have been identified in *M*. *tuberculosis*. It has been proven that these systems help the survival of bacteria under stressful conditions, including starvation, oxidation, immune response, and exposure to antimicrobial agents [3–5].

The TA system consists of two parts: a protein toxin and a cognate antitoxin (protein or nonprotein). The antitoxin controls the toxin [6, 7]. In *M*. *tuberculosis*, the most abundant TA system is type II. Eighty-eight type II families have been identified in *M*. *tuberculosis* H37Rv and play important roles in bacterial survival under different adverse conditions. Type II toxins act as RNases, kinases, and acetyltransferases and are involved in bacterial virulence, biofilm formation, phage inhibition, and various types of stress management, including antibiotic tolerance and persister formation [5]. When the bacterium is exposed to stress conditions such as starvation, hypoxia, and antibiotic exposure, the antitoxin is decomposed, and the toxin affects its target. As a result, the cells in stressful conditions become persistent [5, 8]. The most abundant family within the type II TA systems is *vapBC*. To date, at least 50 *vapBC* families have been identified. Other significant families include 10 *mazEFs*, 3 *relBE* families, 3 *relBE*-like modules (*relBE*, *relFG* and *relJK*), and the tripartite type II TAC (toxin-antitoxin-chaperone) system [5, 8, 9]. Studies have shown that the expression of certain type II families, such as *mazEF3*, *vapBC3*, and *relJK*, is altered in antibiotic-resistant strains. The *mazEF3* family consists of the *mazF3* toxin and its cognate *mazE3* antitoxin. The MazF toxins act as ribosome-independent mRNA endoribonucleases. The toxic effect of MazF in the cell is neutralized by the MazE antitoxin [10]. MazEF plays roles in promoting programmed cell death, inhibition of bacterial growth, persistence of infections, latent TB, and bacterial adaptability to the microenvironment [11]. Additionally, *M*. *tuberculosis* H37Rv contains three RelBE-like modules, *relBE*, *relFG*, and *relJK*, which are expressed in human macrophages during infection [12]. RelK and VapC3 are toxins, both of which have RNase activity, and these toxins are neutralized by RelJ and VapB3, respectively [13]. RelK and VapC3 demonstrate RNase activity, leading to the suppression of colony formation upon overexpression. RelK functions bacteriostatically by impeding bacterial growth and inducing cells to transition into the stationary phase. Interestingly, research indicates that heightened levels of RelK may contribute to a rise in rifampin-tolerant cells [11, 13]. Given that the expression of MazF3, RelK, and VapC3 toxins has been shown to increase in the presence of antibiotics in several studies, this study aims to investigate mutations in these toxins and compare their presence in both rifampin-sensitive and rifampin-resistant strains.

On the other hand, molecular epidemiological studies on *M*. *tuberculosis* infection have shown that drug resistance, tolerance, bacterial adaptation, and different responses to vaccination are related to specific genotypes and/or lineages of *M*. *tuberculosis* [14]. Additionally, recent evaluations have revealed that the type II toxin—antitoxin system varies among different lineages of *M*. *tuberculosis*, with mutations being observed in various lineages [15, 16].

Molecular techniques such as allele-specific oligonucleotide multiplex PCR (ASO-PCR) can be used to classify clinical isolates into genetic groups. Based on phylogenetic relationships, *M*. *tuberculosis* isolates are categorized into 7 main lineages: Indian Ocean (lineage 1), East Asian (lineage 2, which includes the Beijing clade), Indian and East African (lineage 3), Euro-American (lineage 4), West African-1 (lineage 5), West African-2 (lineage 6), and Aethiops vetus (lineage 7) lineages. Studies have indicated that certain lineages of *M*. *tuberculosis*, particularly lineage 2, exhibit increased virulence and antibiotic resistance [14, 17].

Studies have shown that 96% of rifampin-resistant isolates have mutations in the hotspot region of *rpoB*, which encodes the β-subunit of RNA polymerase [18]. Therefore, the present study aimed to investigate the relationships between rifampin resistance and the type of mutation in the *rpo* gene with the lineage and sequence of three important type II genes of the TA system, *mazEF3*, *relJK*, and *vapBC3*.

## Materials and methods

### Sample collection and DNA extraction

Thirty-two rifampin-resistant *M*. *tuberculosis* isolates were collected from four provinces: Tehran, Golestan, Khorasan Razavi, and Semnan. In addition, 121 rifampin-sensitive *M*. *tuberculosis* samples were collected from Golestan Province, which is one of the main centers of tuberculosis in Iran. The required permission to conduct the study was granted by the National Tuberculosis Center and the ethics committee of Golestan University of Medical Sciences. Subsequently, in collaboration with the Infectious Diseases Research Center and the Central Tuberculosis Laboratory of Golestan Province, Iran, access to the samples was authorized. A confirmatory test for rifampin resistance was conducted using the proportional method for all *M*. *tuberculosis* isolates [19]. For molecular evaluation, DNA extraction was carried out using a Cinnagen DNA Extraction Kit (Cinnagen, Co. Iran). The extracted DNA was stored at -20 °C. Subsequently, molecular confirmation of the bacteria and antibiotic resistance to rifampin was performed for all *M*. *tuberculosis* isolates.

### Molecular confirmation of *M*. *tuberculosis* isolates

Molecular confirmation of *M*. *tuberculosis* species was conducted using *IS6110* PCR. The PCR was carried out in a 25 μl volume, consisting of 10x PCR buffer, 1.5 mM MgCl_2_, 10 mM dNTPs, 0.5 μM of each primer, 1.5 U of Taq DNA polymerase, and 100 ng of extracted DNA. The Thermocycler program included initial denaturation at 94°C for 4 minutes, followed by 30 cycles denaturation at 94°C for 30 seconds, annealing at 68°C for 40 seconds, and extension at 72°C for 55 seconds, and final extension at 72°C for 8 minutes. The resulting amplicon size was 123 bp. Positive control sample included *M*. *tuberculosis* H37Rv and distilled water were used as positive and negative controls, respectively [20].

### Evaluation of *rpoB* gene mutations

The confirmation of rifampin resistance was initially conducted using the proportional method for all 153 isolates [19]. In addition, mutations in the *rpoB516*, *rpoB526*, and *rpoB531* genes were assessed for all *M*. *tuberculosis*-resistant isolates. Furthermore, resistance to isoniazid was determined by analyzing mutations in *katG* codon 315 and *mab-inhA*-15 using multiple allele-specific polymerase chain reaction (MAS-PCR) based on the method outlined by Chia et al. The reaction mixture included the following primers: *rpoB516* (1 pmol), *rpoB526* (5 pmol), *rpoB531* (32.5 pmol), *RIRm* (30 pmol), *katG* F (1 pmol), *katG* R (1 pmol), *inhAP*-15 (6 pmol) and *inhAP* F (6 pmol). The other reagents used were 10X PCR buffer (2.5 ml), 50 mM MgCl_2_ (2 ml), 10 mM dNTP mixture (0.5 ml), 5 U/ml Taq Polymerase (0.1 ml), DNA template (20 ng), and PCR-grade water to obtain a final volume of 25 ml. The Thermocycler temperature and time protocol were as follows: 96°C for 3 minutes, 25 cycles of 95°C for 50 seconds, 68°C for 40 seconds, and 72°C for 1 minute, and a final extension at 72°C for 7 minutes. The PCR products were electrophoresed on a 2.5% agarose gel to evaluate of mutations [21]. The absence of bands at 293, 270, 218, 184, and 170 bp indicated the mutations in the *katG*, *mabA-inhA*, *rpoB516*, *rpoB526*, and *rpoB531* gene, respectively.

### Lineage typing of *M*. *tuberculosis* isolates

For lineage typing, the ASO-PCR method designed by Carcelén et al. was utilized, with specific primers listed in Table 1 [22]. The Thermocycler temperature and time protocol were as follows: 95°C for 10 minutes, 29 cycles of 95°C for 1 minute, 62°C for 1 minute, and 72°C for 1 minute, and a final extension at 72°C for 10 minutes. After electrophoresis of the PCR product, the lineage type was determined based on the presence or absence of the amplicon. According to Carcelén et al. study, lineage 1, lineage 2, lineage 3, lineage 4, lineage 5, and lineage 6 lack amplicons of 95 bp, 345 bp, 434 bp, 520 bp, 183 bp, and 267 bp, respectively [22].

**Table 1:** Primers used in this study.

	Primer	Primer Sequence (5’-3’)	Size	Reference
***Molecular confirmation***	*IS6110*	F: CCTGCGAGCGTAGGCGTCGG R: CTCGTCCAGCGCCGCTTCGG	123	[20]
***Lineage typing***	*SNP1*	F: GAGGATGTTCGCGCCGA R: TCCAGCAGCACCACGAC	95	[22]
	*SNP2*	F: TCAACCTGTACCACCGCAC R: CGGCGTATGGGAAGTACCC	345	
	*SNP3*	F: GTTGCATTCCTACGAGTTCACC R: CGCCACGAACCCTGTCA	434	
	*SNP4*	F: CAGCCTTAAGAGCCAGATCCT R: ACCTACCAGCACCGTCATC	520	
	*SNP5*	F: ATCGTTGGCGTGGACCTC R: GAAGAACACCCCGGCCAC	183	
	*SNP6*	F: ATATCGGTTCGGCGGGC R: CGACCGAATGCTTGTACTGC	267	
***MAS-PCR***	*katG* gene (at S315)	F: ATACGACCTCGATGCCGCT R: GCAGATGGGGCTGATCTACG	293	[21]
	*mabA-inhA*:-15	F: CACCCCGACAACCTATCG R: GCGCGGTCAGTTCCACA	270	
	*rpoB* gene (at D516)	F: CAGCTGAGCCAATTCATGGAC (*RIRm*): TTGACCCGCGCGTACAC	218	
	*rpoB* gene (at H526)	F: CTGTCGGGGTTGACCCA R: *RIRm*	185	
	*rpoB* gene (at S531)	F: CACAAGCGCCGACTGTC R: *RIRm*	170	
***Toxin-Antitoxin Analysis***	*mazEF3*	F: TGCCTTTCCACACTTTGACG	825 bp	[23]
		R: CTTCGCTTCTTGGTGCTGAC		
	*relJK*	F: GGTAGCCTTGCCGTCCAG R: GGTGGTACTCGATCAGGTCA	875 bp	
	*vapBC3*	F: GCAATGCGGTCTACTCGTG	934 bp	
		R: TGCATGAAGTCTGGTCCCTC		

### Evaluation of the *mazEF3*, *relJK*, and *vapBC3* genes of type II toxin-antitoxin complexes *in M*. *tuberculosis* isolates

Mutations in the *mazEF3*, *relJK*, and *vapBC3* genes in lineage 2, 3, and 4 of *M*. *tuberculosis* (sensitive and resistant isolates) were investigated using specific primers listed in Table 1 [23]. The *mazEF3*, *relJK*, and *vapBC3* genes were sequenced in 16 isolates (8 rifampin-sensitive and 8 rifampin-resistant isolates). Finally, the relationships of these mutations with protein changes and antibiotic resistance were evaluated.

Among the rifampin-sensitive and resistant *M*. *tuberculosis* isolates belonging to each lineage, certain isolates with varying mutations in the *rpoB* gene were chosen, and their PCR products were sent to the Momgene Company (in Iran) for sequencing. Their sequences were analyzed using the NCBI database, COBALT for protein alignment, and SnapGene 2017. Subsequently, the sequences were submitted to the NCBI website, and assigned accession numbers.

## Results and discussions

### Molecular confirmation of *M*. *tuberculosis* isolates and detection of rifampin resistance

The molecular approach using IS6110 PCR, confirmed the accurate detection of all *M*. *tuberculosis* isolates. All the investigated isolates showed the desired amplicon (123 bp). Among the isolates, 121 were sensitive and 32 were resistant to rifampin, as determined by the proportional method. These isolates were analyzed using the MAS‒PCR method to identify mutations in the *rpoB* hotspot regions. Mutations were only observed in the resistant isolates, confirming their resistance. This molecular approach successfully confirmed the sensitivity and resistance of the *M*. *tuberculosis* isolates.

### Distribution of mutations in the *rpoB* gene

In phenotypically rifampin-sensitive isolates, no gene mutation leading to resistance to rifampin were observed. However, all phenotypically rifampin-resistant isolates had at least one mutation in the hotspot region of the *rpoB* gene. This indicates that mutations in these three codons of the *rpoB* gene directly correlate with phenotypic resistance. Mutations in the *rpoB531* gene were found in 20 isolates (62.5% of the resistant isolates), with 15 samples showing individual mutations and 5 isolates showing mutations in combination with others. Mutations in the *rpoB516* gene were individually observed in 12 patients and in combination with other mutations in 5 samples. Mutation in the *rpoB526* gene was the least frequent and was observed in only one isolate. This mutation occurred simultaneously with other mutations. In the MAS-PCR method, mutations in the *katG* and *mabA-InhA* genes, which are responsible for isoniazid resistance, were also investigated. Four isolates (12.5%) were determined to be isoniazid resistant. These strains are also resistant to rifampin, which is known as MDR.

### Lineage of *M*. *tuberculosis* isolates

Twenty-eight (18.30%), 50 (32.67%), and 75 (49.01%) MTB isolates belonged to lineages 2, 3, and 4, respectively. The frequency of rifampin resistance among these strains was 7 (25%), 9 (18%), and 16 (21.3%), respectively, showing that the resistance in lineage 2 was greater than that in the other lineages (Table 2). The Chi-square test was used to investigate the relationship between lineage type and resistance and sensitivity to rifampin. Although the amount of antibiotic resistance in lineage 2 was higher than that of lineages 3 and 4, a p-value of 0.761 was obtained, indicating the absence of a significant relationship between antibiotic resistance and lineage type. However, in various studies, this relationship has been found to be significant.

**Table 2:** The rate of antibiotic resistance in different lineages of *M*. *tuberculosis*.

Lineages	Sensitive isolates	Resistant isolates	Total
**Lineage 2**	21 (75%)	7 (25%)	28 (100%)
**Lineage 3**	41 (82%)	9 (18%)	50 (100%)
**Lineage 4**	59 (78.7%)	16 (21.3%)	75 (100%)
**Total**	121	32	153

Among all the samples, 4 isolates (12.5%) were considered multidrug-resistant (MDR), and all of them belonged to lineage 2. In this lineage, none of the rifampin-resistant isolates had a single mutation in *rpoB516*, and 5 isolates (71.4%) had simultaneous mutations. However, simultaneous mutations were not found in members of lineages 3 and 4 (Table 3).

**Table 3:** Mutation rates of the investigated genes in the different lineages.

Mutation	Lineage 2	Lineage 3	Lineage 4
***rpoB516*** (12 isolates: 37.5%)	-	5	7
***rpoB531*** (15 isolates: 46.9%)	2	4	9
***rpoB516*, *rpoB531*, *mabA-inhA*** (3 isolates: 9.4%)	3	-	-
***rpoB516*, *rpoB531*** (1 isolate: 3.1%)	1	-	-
***rpoB516*, *rpoB526*, *rpoB531*, *mabA-inhA*** (1 isolate: 3.1%)	1	-	-
**Total** (32 Resistant isolates)	7	9	16

### Toxin-antitoxin system analysis

The genes *mazEF3*, *relJK*, and *vapBC3* were sequenced in 16 isolates half of which were rifampin-sensitive and the other half rifampin-resistant. The accession numbers of these samples in GenBank are provided in Table 4.

**Table 4:** Accession numbers of TA systems of 16 MTB isolates in the NCBI database.

Species	Type	Lineage	Strain	Accession Number		
				MazEF3	RelJK	VapBC3
***M*. *tuberculosis***	**Resistant Isolates**	L2	TR1	OR268987.1	OR269015.1	OR269043.1
		L2	TR2	OR268988.1	OR269016.1	OR269044.1
		L3	TR3	OR268989.1	OR269017.1	OR269045.1
		L3	TR4	OR268990.1	OR269018.1	OR269046.1
		L3	TR5	OR268991.1	OR269019.1	OR269047.1
		L4	TR6	OR268992.1	OR269020.1	OR269048.1
		L4	TR7	OR268993.1	OR269021.1	OR269049.1
		L4	TR8	OR268994.1	OR269022.1	OR269050.1
	**Sensitive Isolates**	L2	TS1	OR268995.1	OR269023.1	OR269051.1
		L2	TS2	OR268996.1	OR269024.1	OR269052.1
		L3	TS3	OR268997.1	OR269025.1	OR269053.1
		L3	TS4	OR268998.1	OR269026.1	OR269054.1
		L3	TS5	OR268999.1	OR269027.1	OR269055.1
		L4	TS6	OR269000.1	OR269028.1	OR269056.1
		L4	TS7	OR269001.1	OR269029.1	OR269057.1
		L4	TS8	OR269002.1	OR269030.1	OR269058.1

**Fig 1:**
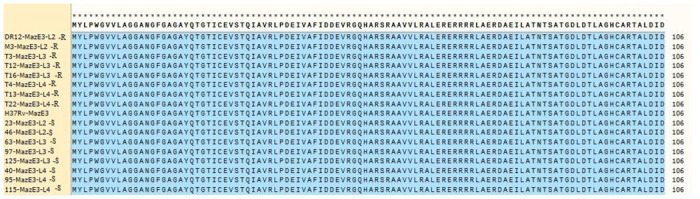
MazE3 (antitoxin) amino acid sequence; SnapGene 2017. S: Sensitive isolates, R: Resistant isolates. Blue: no mutation.

**Fig 2:**
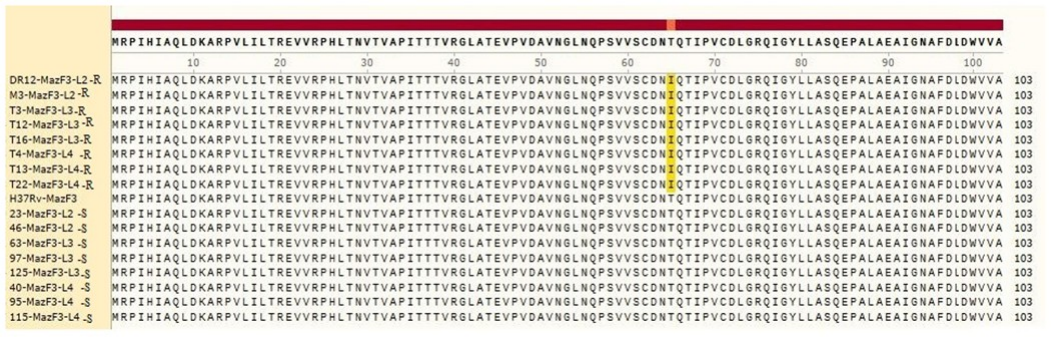
MazF3 (toxin) amino acid sequence; SnapGene 2017. S: Sensitive isolates, R: Resistant isolates. White: no mutation; yellow: mutation.

Sequence analysis in SnapGene 2017 software revealed that the *mazE3* gene and its corresponding protein, MazE3 antitoxin, were similar in all investigated isolates across the three lineages. Comparison of these sequences with the standard sequence of *M*. *tuberculosis* H37Rv did not show any mutations. However, the *mazF3* toxin gene in lineage 3 rifampin-resistant isolates had a mutation at nucleotide 473, changing the nucleotide G to A without altering the amino acid sequence. In all rifampin-resistant isolates, regardless of lineage, a mutation occurred at codon 65 of the toxin gene, changing the nucleotide C to T and resulting the amino acid T (threonine) changing to I (isoleucine). Figs 1 and 2 display the protein sequences of the antitoxin (MazE3) and toxin (MazF3), respectively.

**Fig 3:**
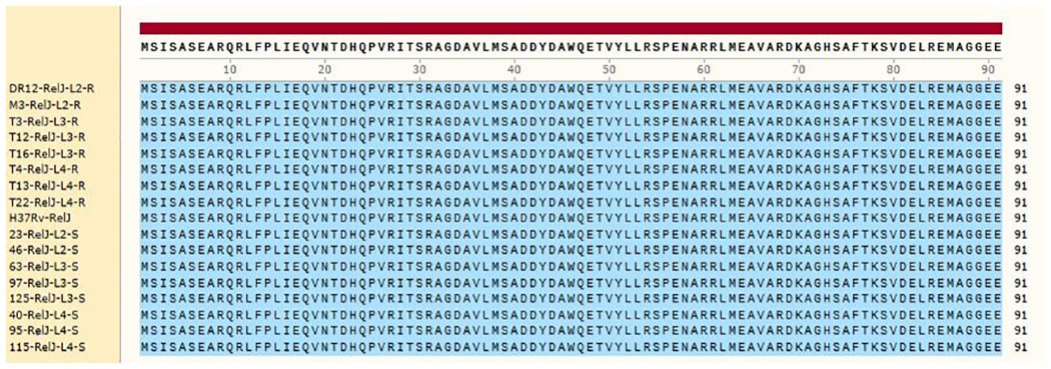
RelJ (antitoxin) amino acid sequence; SnapGene 2017. S: Sensitive isolates, R: Resistant isolates. Blue: no mutation; yellow: mutation.

**Fig 4:**
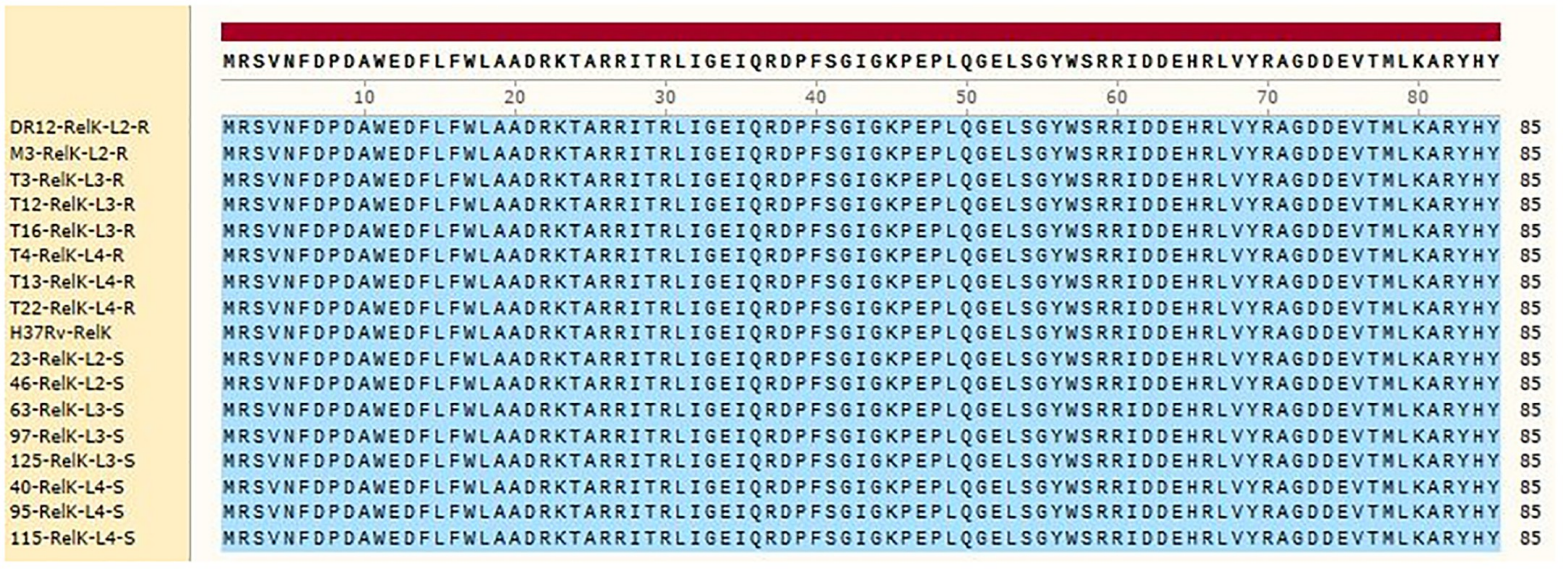
RelK (toxin) amino acid sequence; SnapGene 2017. S: Sensitive isolates, R: Resistant isolates. Blue: no mutation; yellow: mutation.

Compared to the standard sequence of *M*. *tuberculosis* H37Rv, the resistant isolates in lineage 3 had a mutation at nucleotide 237 in the *relJ* gene where the nucleotide T was changed to G, but the amino acid sequence remained unchanged. Figs 3 and 4 shows the antitoxin and toxin, respectively. In comparison to the *M*. *tuberculosis* H37Rv sequence, the toxin sequence (*relK*) does not contain any mutations.

**Fig 5:**
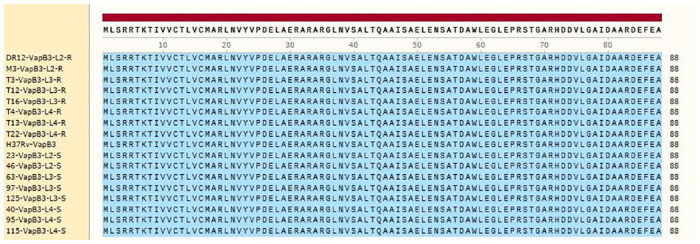
VapB3 (antitoxin) amino acid sequence; SnapGene 2017. S: Sensitive isolates, R: Resistant isolates. Blue: no mutation; yellow: mutation.

**Fig 6:**
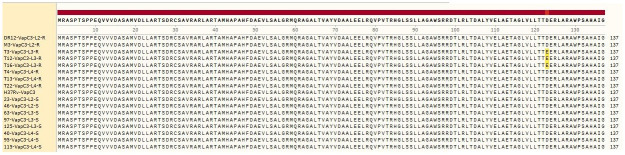
VapC3 (toxin) amino acid sequence; SnapGene 2017. S: Sensitive isolates, R: Resistant isolates. White: No mutation; Yellow: Mutation.

Sequence analysis of VapB3 (antitoxin) using SnapGene software revealed that this sequence has no mutations compared to the standard sequence of *M*. *tuberculosis* H37Rv. VapC3 consists of 137 amino acids, and the resistant isolates of lineage 3 exhibit a mutation in codon 123 (nucleotide 369), where the nucleotide C is changed to G, resulting in a changed from amino acid D (aspartic acid) to E (glutamic acid). Figs 5 and 6 depict VapB3 (antitoxin) and VapC3 (toxin), respectively.

In total, the evaluation of the *mazEF3*, *relJK*, and *vapBC3* gene sequences of the type II toxin-antitoxin system in *M*. *tuberculosis* revealed that the toxin sequences in lineage 3 had mutations in all three families (Table 5). Specifically, in the *vapC3* toxin, this mutation resulted in an amino acid change from aspartic acid to glutamic acid.

**Table 5:** Mutations in the *mazEF3*, *vapBC3*, and *relJK* genes in different lineages of rifampin-resistant *M*. *tuberculosis* isolates.

Toxin-Antitoxin family		Lineage 2	Lineage 3	Lineage 4
**MazEF3**	**MazE3**	No Mutation	No Mutation	No Mutation
	**MazF3**	Codon 65 (C to T): Threonine to Isoleucine	Codon 65 (C to T): Threonine to Isoleucine Nucleotide 473: G to A	Codon 65 (C to T): Threonine to Isoleucine
**RelJK**	**RelJ**	No Mutation	Nucleotide 237: T to G	No Mutation
	**RelK**	No Mutation	No Mutation	No Mutation
**VapBC3**	**VapB3**	No Mutation	No Mutation	No Mutation
	**VapC3**	No Mutation	Nucleotide 369: C to G Aspartic acid to Glutamic acid	No Mutation

Tuberculosis remains a significant global health issue. Over the past 50 years, Iran’s health authorities have made significant progress in controlling tuberculosis. As a result, the incidence of tuberculosis has decreased from 142 cases per 100,000 people in 1964 to 12.6 cases in 2016 [24, 25]. Despite Iran’s proximity to countries such as Azerbaijan, Pakistan, Afghanistan, and Iraq, as well as high rates of immigration, the incidence of drug-resistant tuberculosis in Iran is lower than expected, ranging from 0–2.9% [26].

Genomic mutations including SNPs, small insertions, or deletions, and occasionally larger deletions or inversions, are major contributors to antibiotic resistance in *M*. *tuberculosis*. Due to absence of horizontal gene transfer or episomal resistance genes, these mutations typically occur spontaneously and are encoded in chromosome. The spread of resistant bacteria occurs through replication within the host and onward transmission between hosts. Although horizontal gene transfer is absent in *M*. *tuberculosis*, target-based mutations, activator mutations, and modulation of efflux pumps are major mechanisms for acquiring antituberculosis drug resistance [27–29].

In addition to these factors, several elements play a role in the development of antibiotic resistance, including the phylogenetic lineage of *M*. *tuberculosis*, genetic modules, metabolic adaptation to the environment, and host-specific factors. Furthermore, specific lineages of *M*. *tuberculosis* may exhibit high mutation rates and a strong ability to acquire drug resistance. Several studies have demonstrated that the drug resistance of the Beijing clade from lineage 2 (East Asian Lineage) was higher than that of other lineages [30]. This study also investigated the mutations in the *mazEF3*, *vapBC3*, and *relJK* families of the type II toxin-antitoxin system in both resistant and sensitive isolates, with the goal of determining the differences in the gene structure of these families between these isolates.

In this study, only three lineages (2, 3, and 4) of *M*. *tuberculosis* were found in various regions of Iran, among both drug-sensitive and drug-resistant isolates. Lineage 4 was the most prevalent lineages among rifampin-sensitive isolates, while lineage 2 was most common among rifampin-resistant isolates. Although the incidence of resistance was higher in lineage 2 (25%), in the present study, this difference is not significant in sensitive and resistant isolates of the lineages (P>0.05).

The diversity of *M*. *tuberculosis* strains in Iran, based on family/subspecies, showed that New-1 (L4) had the highest frequency, at 21.94%. CAS (L3) with 19.21%, EAI (L1) with 12.95%, and T (L4) with 12.16% were the dominant circulating *M*. *tuberculosis* genotypes, while lineages 5, 6 and 7 were not reported from Iran. Additionally, the observation of lineage 3 as one of the common subpopulations of *M*. *tuberculosis* in Iran may reflect the characteristics and pathogenic potential of this genotype. Recently, the incidence of MDR strains in lineage 3 in the world has been estimated to be 30.63% [31]. However, this amount is estimated at 21.1% in Iran [32]. In a study by Babaii Kochkaksaraei et al. in Golestan Province, which is the second center of tuberculosis in Iran, lineage 4 (36%), lineage 2 (22%) and lineage 3 (15%) had the highest frequencies. The greatest amount of antibiotic resistance was observed in lineage 2 [33].

In the study by Mansoori et al. in Golestan Province, one-third of the studied strains were found to be related to lineage 3, which had primarily migrated to this province from Sistan and Baluchistan [34]. This finding highlights the importance of conducting additional research and monitoring of this subpopulation.

In *M*. *tuberculosis*, resistance to rifampin is primarily linked to resistance to isoniazid, categorizing it as MDR. Another objective of this study was to identifying the specific mutations and their frequency in the key genes responsible for rifampin resistance (*rpoB531*, *rpoB516* and *rpoB526*). All rifampin-resistant *M*. *tuberculosis* isolates exhibited at least one mutation in the *rpoB* hotspot region. Among these, the *rpoB531* mutation was the most common in the rifampin-resistant isolates (62.5%), consistent with findings from other studies [35, 36]. In studies conducted in Iran, the prevalence of *rpoB531* was reported to be 40% in Tehran and 26% at the border of Afghanistan [37]. This mutation was detected in 53% of Brazil, 59.83% of North India, 58% of Thailand, 60% of India, 53% of Greece, and 59% of Italy [38–43].

Although the prevalence of rifampin resistance in lineage 2 members is higher than in other lineages, our results showed that there is no relationship between rifampin resistance and the lineage of *M*. *tuberculosis*. One important finding in our study is that multiple mutations in the *rpoB* gene were only found in lineage 2.

Several studies have investigated the role of the type II toxin—antitoxin system in the emergence of antibiotic resistance in the presence of antibiotics. Keren et al. (2011) reported that drug-resistant isolates show increased expression levels of certain TA systems under antibiotic stress conditions. These systems include VapC3/VapB3, Rv2021c/Rv2022c, Rv1989c/Rv1990c, RelF antitoxin/RelG toxin, Rv0918/Rv0919, Rv3180c/Rv3181c, higB/higA, Rv3188/Rv3189, and Rv2034/Rv2035. The authors also demonstrated that the expression of higB/higA, Rv2034/Rv2035 and Rv2022c increased under starvation and hypoxia conditions [1]. Additionally, Singh et al. showed that the expression level of RelE2 significantly increased in the presence of rifampin, and the expression level of RelE3 increased with gentamicin, levofloxacin, and isoniazid [44].

In several other studies it was found that certain toxins, such as the MazF family, Rv1577c, Rv2651c, Rv0366c, and RelE, are responsible for specific antibiotic tolerance and resistance [27, 28]. Therefore, the present study examined mutations in three families (*mazEF3*, *relJK*, and *vapBC3*) of the type II toxin-antitoxin system in lineages 2, 3, and 4 of *M*. *tuberculosis*. No mutations were found in *vapB3*, *mazE3*, or *relK* in any of the sensitive or resistant isolates, regardless of their lineage, and the sequences of these genes were similar to each other and the H37RV strain.

However, lineage 3 of the *M*. *tuberculosis*-resistant isolates had mutations in nucleotide 369 of the *vapC3* gene. This mutation changed the amino acid from aspartic acid to glutamic acid. Both glutamic acid and aspartic acid are classified as acidic amino acids. Additionally, a nonsense mutation was detected at nucleotide 473 of the *vapC3* gene.

On the other hand, all the rifampin-resistant *M*. *tuberculosis* isolates of lineage 3 had a nonsense mutation at nucleotide 237 of the *relJ* gene. The role of this gene has not been determined, but it could provide a basis for future studies.

MazEF consists of an antitoxin (MazE) and a toxin (MazF), which form a complex under normal conditions. Previous studies have shown that the *mazE* and *mazF* genes are differentially expressed in drug-sensitive and drug-resistant bacteria [11, 45]. In a study conducted in 2021 by Kazemian et al., it was found that the expression of MazF3 and MazF6 was significantly higher in resistant strains compared to sensitive strains [46]. In the present study, the *mazF3* (toxin component) was mutated in all rifampin-resistant *M*. *tuberculosis* isolates. The mutation occurred at codon 65, where threonine was changed to isoleucine. Threonine and isoleucine are amino acids have two stereogenic centers [47]. Additionally, another nonsense mutation at nucleotide 473 was observed in the *mazF3* gene.

Therefore, although the highest level of resistance to rifampin in *M*. *tuberculosis* was observed in lineage 2, the highest level of mutation in the three gene operons encoding the toxin-antitoxin system was observed in lineage 3. In the Solano-Gutierrez study, a total of 65 mutations were observed in Lineage 4 and 28 mutations in Lineage 3. Protein MazF8, VapC49, MazE2, and VapC24 proteins had amino acid deletions, while Rv0918, Rv2653c, VapC25, VapC49, and Rv0836c showed several amino acid changes. Additionally, mazF3, vapC47, vapC6, and vapC10 were identified as new biomarkers suitable for genotyping [15]. These findings suggest the diversity of genes affecting resistance to rifampin in different parts of the world and can serve as the basis for further research on the toxin—antitoxin system and its impact on rifampin resistance.

On the other hand, a study conducted by Zaychikova et al observed that certain genes in type II TA systems have SNPs associated with specific genotypes. Consequently, a set of genes in TA systems was proposed to classify *M*. *tuberculosis* into nine main genotypes. The gene set included *higA1*, *vapC6*, *vapC10*, *vapC38*, *mazF3*, *mazF8*, and *vapC47* genes [48]. Additionally, in Solano-Gutierrez’s study, the *mazF3*, *vapC47*, *vapC6* and *vapC10* genes were identified as suitable markers for genotyping of *M*. *tuberculosis* isolates [15]. Our results show that the *mazF3* gene can be used to identify resistant isolates because, in our study, regardless of the lineage type, all resistant isolates have a mutation in codon 65. Therefore, in our study, *mazF3* can be used as a new biomarker for identification of resistant isolates, and our findings are consistent with Zaychikova’s and Solano-Gutierrez’s studies. However, it is suggested that more isolates should be investigated in future studies. It is also suggested that future studies investigate the effect of this mutation on its expression and antibiotic resistance.

## Conclusion

In 153 isolates investigated, Lineage 4 (49%), Lineage 3 (7.32%), and Lineage 2 (3.18%) were the most frequent. Among the 32 resistant isolates, rifampin resistance was highest in Lineage 2 (25%), followed by lineage 4 (21%). The most common mutation in rifampin-resistant isolates of *M*. *tuberculosis* was the *rpoB531* gene mutation (5.62%). In lineage 2, unlike other lineages, mutations were mainly observed in multiple points in the *rpoB* gene. Analysis of the *mazEF3*, *relJK*, and *vapBC3* genes structure showed a distinct difference between resistant and sensitive strains in *mazF3*, suggesting a potential link to resistance.

Since only the mutations in these genes were examined in the present study, it would be beneficial to also analyze the expression of these genes, especially *mazF3*, in both sensitive and resistant strains. The *mazF3* gene is specifically mutated in resistant isolates of *M*. *tuberculosis*. Therefore, it is recommended to increase the sample size to conduct a more comprehensive investigation of *mazF3*. If successful, it could potentially be used as a biomarker for developing new methods to identify resistant isolates.
